# Cardiac Contractility Modulation for Heart Failure: Current and Future Directions

**DOI:** 10.1016/j.jscai.2023.101176

**Published:** 2023-12-04

**Authors:** Daniel C. Pipilas, Alan Hanley, Jagmeet P. Singh, Theofanie Mela

**Affiliations:** a Cardiology Division, Massachusetts General Hospital, Boston, Massachusetts; b Harvard Medical School, Boston, Massachusetts; c Demoulas Center for Cardiac Arrhythmias, Massachusetts General Hospital, Boston, Massachusetts

**Keywords:** cardiac contractility modulation, cardiac devices, cardiomyopathy, electrophysiology, heart failure

## Abstract

Cardiac contractility modulation (CCM) is a Food and Drug Administration-approved device-based therapy for patients with heart failure. The system delivers biphasic electric stimulation to the ventricular myocardium during the absolute refractory period to augment left ventricular contraction. CCM therapy promotes acute and chronic changes at the cellular level, leading to favorable remodeling throughout the myocardium. CCM improves quality of life, New York Heart Association class, left ventricular ejection fraction, peak oxygen uptake, and the composite end point of cardiovascular death and heart failure hospitalizations. This review will focus on the biological basis, indications, and evidence for CCM, as well as the future applications of this technology.

## Highlights

• Cardiac contractility modulation (CCM) is indicated in patients with NYHA class III and LVEF 25% to 45%.
• CCM provides excitatory stimulation to augment myocardial contraction.
• Enhanced calcium handling and reverse remodeling at the tissue level.
• Improves quality of life, and the composite of cardiovascular death and heart failure hospitalization.

## Central Illustration

**Figure:**
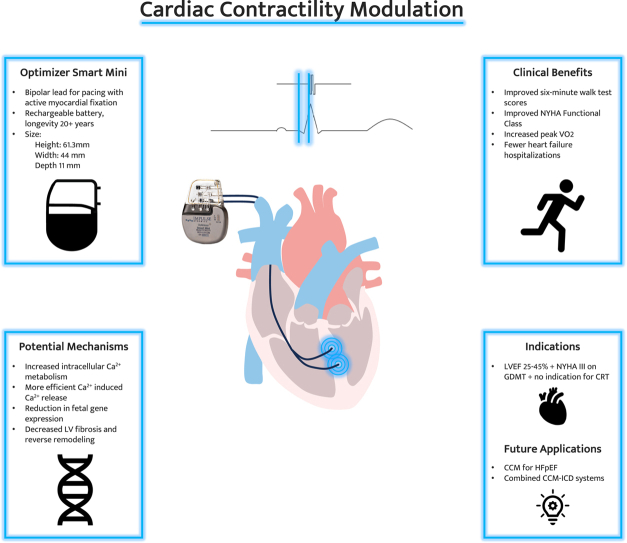

## Introduction

Heart failure (HF) is a complex clinical syndrome that currently affects an estimated 6 million adults in the United States, with that number expected to rise in the coming decades.^1^^,^^2^ HF is a leading cause of mortality and is projected to cost at least $70 billion annually in the United States by 2030.^3^ HF can arise from numerous underlying pathophysiologic processes that impact cardiac structure or function.^4^ Three major HF classifications exist and are defined by left ventricular ejection fraction (LVEF): heart failure with a reduced ejection fraction (HFrEF, LVEF ≤40%), heart failure with mildly reduced ejection fraction (HFmrEF, LVEF 41%-49%), and heart failure with a preserved ejection fraction (HFpEF, ≥50%).^5^ Patients with HF are further characterized by American Heart Association (AHA) and American College of Cardiology (ACC) guideline stage (A-D), which helps characterize the severity of the underlying disease state, and by New York Heart Association (NYHA) classification (I-IV) which delineates the extent of HF symptoms.^5^

Treatment modalities for patients with HF include medications, device-based therapies, and transplantation, but there exists substantial heterogeneity in treatment options available to individual patients within these categories owing both to the breadth of disease processes that cause clinical HF and patient characteristics (eg, age, patient comorbidities, LVEF, QRS-complex width).

Guideline-directed medical therapy is the cornerstone of therapy for patients with HFrEF and has improved substantially in the past 3 decades leading to improved morbidity and mortality in this population.^5^ These medications target adverse neurohormonal activation that occurs as a result of HF and add cumulative benefit to survival. Prospective data in patients with HFmrEF is less robust, and similarly, only sodium-glucose cotransporter-2 (SGLT2) inhibitors are shown to decrease hospitalizations and cardiovascular mortality in the HFpEF population.^6^

In addition to medical therapy, disease-modifying device-based therapies that target the cardiac conduction system improve outcomes in select patients with HFrEF. Although implantable cardioverter-defibrillator (ICD) therapy is indicated in HFrEF patients for primary and secondary prevention of sudden cardiac death, these devices do not modify the disease substrate and thus will not be addressed here.

Normal electrical activation of the heart is uniform, and rapid, and facilitates coordinated contraction of the right and left ventricular (LV) myocardium. In HFrEF, structural and mechanical changes lead to conduction system disease that alters this uniformity and can lead to ventricular dyssynchrony and progressive adverse remodeling. CRT, also termed biventricular pacing, facilitates simultaneous pacing of the right and left ventricles via leads placed in the right ventricular (RV) septum and coronary sinus. CRT has been shown to reverse deleterious cellular, tissue, and organ-level dysfunction that occurs in HF by inducing molecular, genetic, and functional changes.^7^^,^^8^ At the organ level, CRT restores ventricular synchrony, and has been shown to improve mortality, hospitalizations, symptoms, and quality of life in patients with HFrEF and HFmrEF who have a wide QRS (≥150 ms), and select patients with QRS duration between 120 to 149 ms or those with a high pacing burden (eg, patients with atrial fibrillation [AF] who undergo atrioventricular nodal ablation).^5^^,^^9^ In patients with HF and QRS duration <130 ms, CRT is not recommended and may increase mortality.^10^ These strict indications limit who is eligible for CRT and thus only a subset of patients with HFrEF meet criteria for CRT.^11^^,^^12^ Furthermore, roughly 30% of patients respond poorly to CRT and derive minimal benefit.^13^^,^^14^

Neuromodulation via baroreceptor activation is an additional device-based therapy that is Food and Drug Administration (FDA)-approved to treat patients with HFrEF and is indicated in patients who are not candidates for CRT.^15^ Baroreceptor activation therapy targets autonomic imbalance and has shown improvement in exercise capacity and quality of life, but there is limited data on mortality and hospitalizations. Despite the widespread availability of CRT and the growing use of neuromodulation, many patients are not eligible for these treatments and are left without options for device-based therapy. Thus, significant gaps in treatment options exist for patients with HFrEF and HFmrEF with narrow QRS on optimal medical therapy (OMT), as well as those with HFpEF. Accruing evidence suggests that these populations may derive benefit from CCM.

In 1969, it was shown that excitatory stimulation applied during the absolute refractory period augments myocardial contraction.^16^ This observation served as the basis for the development of CCM therapy in HF to augment systole and provide a positive inotropic effect to failing hearts.^17^^,^^18^ The CCM system uses standard pacing electrodes to deliver high-voltage, nonexcitatory impulses during the absolute refractory period and is implanted in a procedure similar to permanent pacemaker (PPM) and ICD insertions.^19^ Initial studies in preclinical HF models showed improvement in cardiac contractile parameters and ventricular function,^20^, ^21^, ^22^ and early human studies showed improvement in LVEF, symptoms, and functional status.^23^. CCM has been studied in the HF population for over 2 decades, and this therapy is the subject of significant interest for the treatment of all patients with HF regardless of LVEF and QRS duration. The device was first approved for use in Europe in 2016. Two seminal clinical trials, FIX-HF-5^24^ and FIX-HF-5C,^25^ later resulted in the 2019 breakthrough FDA approval of the Optimizer Smart System CCM device (Impulse Dynamics Inc) for patients with NYHA class III HF with LVEF of 25% to 45%. This review will summarize the physiologic basis for CCM, technical aspects of the therapy, and available evidence for use in HF patients (Central Illustration).

**Central Illustration:**
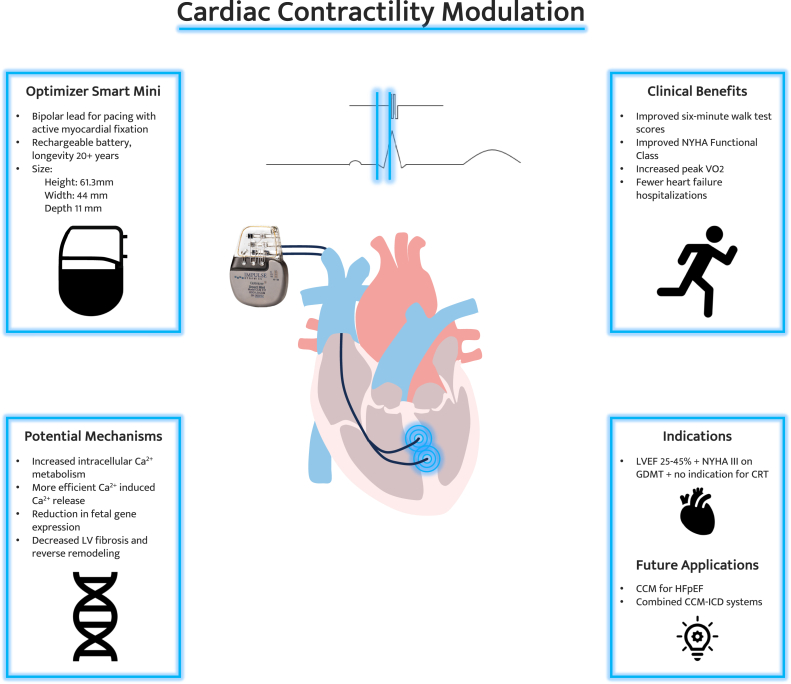
**Cardiac contractility modulation.** Device specifications for the Optimizer Smart Mini device, clinical benefits, potential mechanisms, and approved indications are shown. Acknowledgement: Giorgio A. Medranda, MD, constructed the final Central Illustration.

## Physiologic mechanism of action of cardiac contractility modulation

This section will highlight major insights into the physiologic rationale for cardiac contractility modulation (CCM) therapy. Mechanisms underlying the benefit of CCM are multifactorial. In general, an acute effect on cellular calcium handling and influence on gene expression lead to regional and global improvement in myocardial contractility without an increase in myocardial oxygen demand.

At the cellular level, contraction of the myocardium begins with plasma membrane depolarization, resulting in calcium entering the cytosol from extracellular and intracellular stores.^26^ This influx of calcium leads to muscle contraction through subsequent binding of calcium ions to troponin, resulting in the sliding of contractile proteins actin and myosin.^27^ The magnitude and duration of this rise in intracellular calcium is a major regulator of the force of myocardial contraction.^27^ In failing hearts, adverse neurohormonal activation results in distorted intracellular calcium handling and impairs force production and relaxation.^28^ Over time, metabolic stress, inflammation, altered ion channel expression, reduced function of contractile proteins, and deleterious gene expression lead to progressive myocardial dysfunction and worsening of clinical HF. Impaired calcium handling occurs via several mechanisms including downregulation of genes related to calcium cycling, upregulation of myocardial fetal and stretch response genes, reduced L-type calcium channel (LTCC) activity, decreased sarcoplasmic reticulum calcium uptake and release, and enhanced clearance of calcium by the sodium-calcium exchange protein 1.^17^ These cellular changes correspond with progressive ventricular dilation, eccentric and concentric hypertrophy, tissue fibrosis, and electrical and mechanical dyssynchrony at the organ level, leading to further remodeling.

The mechanism by which CCM improves functional status is likely related to changes in calcium handling and correction toward more normal myocardial gene expression and calcium regulation, and over time these effects occur throughout the ventricular myocardium and contribute to reverse LV remodeling.^19^ Early preclinical studies first demonstrated that high-amplitude CCM pulses applied to ferret hearts during the absolute refractory period increased the amplitude and decreased the time-to-peak values of cytoplasmic calcium currents.^20^ These changes correlated with changes in muscle contraction. Several mechanisms have been proposed for the increase in cytoplasmic calcium with CCM, which include an increased activation and duration of opening of ryanodine receptors, increased calcium influx via LTCC, and increased calcium influx into the sarcoplasmic reticulum via the sarcoplasmic reticulum calcium adenosine-triphosphatase-2a (SERCA2a).^17^ In 1 study of canines with chronic HF, 1 hour of CCM therapy resulted in upregulation of LTCC and improved calcium-induced calcium release.^22^

In addition to short-term effects related to calcium handling, long-term CCM therapy upregulates genes related to calcium cycling and handling, reduces fetal gene expression, and results in reverse remodeling at the tissue level.^29^, ^30^, ^31^ One study of biopsy specimens from patients with HF receiving CCM therapy for 3 months showed increased expression of SERCA2a, phospholamban, and RyR2, indicating favorable changes in gene expression.^31^ The global effect of CCM on LV contractility and reverse remodeling was shown in a study by Yu et al, in which 30 patients undergoing CCM therapy for 3 months demonstrated reduced LV end-systolic volume, improvement in LVEF, and global improvement in myocardial contraction by real-time 3-dimensional echocardiography.^30^ Despite these positive inotropic effects, myocardial oxygen demand does not seem to increase with CCM,^31^, ^32^, ^33^ perhaps because CCM acts through mechanisms independent of cyclic adenosine monophosphate and improves the relationship between oxygen consumption and myocardial workload.^17^

## Device description and technical considerations

Several CCM systems have been developed, but as of 2023, the Optimizer Smart system and the Optimizer Smart Mini system are the only 2 devices that are FDA-approved. The Optimizer system consists of a pulse generator unit implanted subcutaneously in the upper chest wall and 2 ventricular leads embedded in the RV septum. The 2 leads are referred to as the RV lead, defined by the earliest ventricular sensed event, and the local sense (LS) lead. Note that earlier CCM technology required an atrial lead, but the safety and efficacy of a system consisting of only ventricular leads was demonstrated in the FIX-HF-5C2 study; thus, systems containing atrial leads will not be described here.^34^ The Optimizer device is similar in size to a dual-chamber PPM and is recharged on a weekly basis by the patient with an estimated longevity of 15 to 20 years before replacement is required. The CCM system provides therapy for 5 to 12 hours each day (programmed at physician discretion) in 1-hour intervals dispersed evenly over 24 hours. Of note, longer duration of therapy has not been shown to improve outcomes.^35^

The implantation procedure of these devices is similar to that of a dual-chamber PPM and the complications of device insertion and overall complication rate are similar to those of PPM and ICD insertions.^25^^,^^34^^,^^36^ A detailed guide to the CCM implantation was published in 2017 by Kuschyk et al and outlines the insertion procedure, testing protocol, and special considerations.^37^ Briefly, the procedure is typically performed in a sterile, fluoroscopy-enabled electrophysiology laboratory or operating room.^36^ Site selection in the left or right upper chest wall depends on the presence of a pre-existing PPM or ICD (usually in the left chest wall), and the Optimizer system is implanted on the contralateral side. Vascular access is obtained via the axillary and/or subclavian veins for insertion of 2 guide wires that will be used to guide the leads into the RV septum. The leads should be at least 2 cm apart and inserted into the septomarginal trabeculations in the inferior portion of the septal RV outflow tract. Prior to extending the fixation screws at the tip of the leads, an adequate ventricular waveform should be confirmed as excellent sensing is a priority to ensure appropriate device function. Blunt dissection is used to construct a pocket in the prepectoral area. Leads are secured into the pocket, and further programming and testing are conducted.

Proper programming of the device is critical to ensure delivery of CCM stimulation at precisely the right time in each cardiac cycle. Inadvertent firing could result in unintended ventricular pacing or arrhythmia generation.^38^ The 2 leads are programmed to sense ventricular activation events in time with respect to one another. Once a ventricular event is detected by the RV lead, the local sense alert window is opened which represents the delay between the sensed RV event and the sensed local sense event. The length of this window is programmable and tells the device how to discriminate between normal beats and abnormal beats (eg, premature ventricular contractions). Normal beats that fall within the local sense alert window result in CCM delivery, which is usually 30 to 40 ms after the QRS complex. Although the energy delivered by CCM is several times greater than the energy delivered by a pacemaker (∼7.5V), myocardial depolarization does not occur because the CCM stimulus is delivered during the absolute refractory period. Following the completion of testing, the pocket is closed in layers.

Certain technical concerns must be considered in patients undergoing insertion of an Optimizer system. First, if a patient has another implanted cardiac device in place such as an ICD, it is critical to test for device-device interactions. The large voltage signal delivered by the CCM can be inappropriately interpreted as a ventricular arrhythmia by an ICD. Similarly, in patients with PPM, it is critical to ensure that CCM pulses do not inhibit RV pacing. Furthermore, in patients with indwelling devices, insertion of 2 additional leads through the tricuspid valve may worsen tricuspid regurgitation and may increase infection risk. Importantly, it is also possible for patients to experience chest discomfort given the large electrical current delivered via CCM. Therefore, it is important to test the therapy while the patient is awake before securing the leads. If a patient experiences chest discomfort, the leads must be repositioned. Finally, patients with mechanical tricuspid valves or lack of adequate venous access (eg, venous occlusion) are not eligible for CCM given issues with lead placement.

## Clinical efficacy and available evidence

CCM has been studied extensively in the HF population (Table 1).^24^^,^^25^^,^^30^^,^^34^^,^^35^^,^^39^, ^42^, ^43^, ^41^, ^45^, ^46^, ^47^, ^48^, ^49^, ^50^, ^51^, ^52^ In 2004, the observational FIX-HF-3 study demonstrated encouraging safety and efficacy results in 23 patients with improvement in LVEF, quality of life by Minnesota Living with HF Questionnaire (MLWHFQ)^52^ score, and an increased 6-minute walk test.^39^ This gave way to the first randomized study, FIX-HF-4, which was published in 2008.^42^ In this randomized, double-blind, crossover study 164 patients with <35% and NYHA class II or III symptoms on OMT underwent placement of a CCM pulse generator and either received CCM for 3 months followed by sham treatment for 3 months, or sham treatment for 3 months followed by CCM for 3 months.^42^ During the first 3 months, peak myocardial oxygen consumption (VO_2_) increased in both groups, but during the second phase, peak VO_2_ decreased in the group switched to sham and increased in the group switched to CCM. Quality of life by MLWHFQ trended better with treatment during the first 3 months, increased during the second 3 months in the group switched to sham, and decreased further in patients switched from sham to active CCM. Overall, the group that switched from sham (first 3 months) to CCM (second 3 months) had statistically significant improvements in MLWHFQ and peak VO_2_ compared to the group switched from CCM to sham. The study was underpowered to detect differences in mortality or HF admissions, but the improvement in functional status observed with CCM therapy confirmed prior observation and formed the basis for further prospective investigation.

**Table 1:** Selected studies of cardiac contractility modulation.

Study	n	Follow-up	Clinical question	Randomized (Yes/No)	Comparison group	Primary outcome	Additional findings
Stix et al,^39^ 2004 (FIX-HF-3)	23	8 wk	Feasibility of CCM in patients with LVEF ≤35% and NYHA III	No	Baseline	LVEF increased from 22 ± 7% to 28 ± 8%, *P* = .0002	MLWHFQ and 6MWT also improved significantly
Neelagaru et al,^40^ 2006	49	6 mo	Pilot study for FIX-HF-5 in patients on OMT with LVEF ≤35% and NYHA III-IV	Yes	Control	Primary safety end point met, no difference in survival free of any hospitalization (HR, 0.47; 95% CI, 0.16-1.40)	Nonsignificant improvement in 6MWT and peak VO_2_ in treatment compared to control
Nägele et al,^41^ 2008	16	3 mo	Assess CCM in CRT nonresponders on OMT with NYHA III-IV	No	Baseline	CCM implantation is feasible in CRT nonresponders, no electrical interference events between CRT and CCM	Significant increase in NYHA class and LVEF following CCM implantation
Borggrefe et al,^42^ 2008 (FIX-HF-4)	164	6-mo crossover (3 mo of CCM)	Efficacy of CCM in patients with LVEF ≤35% and NYHA II, III, IV	Yes	Active treatment vs sham	Improved peak VO_2_ on treatment (0.52 ± 1.39 mL/kg/min; 95% CI, 0.04-0.99; *P* = .032) and MLWHFQ on treatment (2.93 ± 8.01, ;95% CI, 0.29-5.56; *P* = .03 on treatment)	—
Yu et al,^30^ 2009	30	3 mo	Effect of CCM on LV function using echocardiography	No	Baseline	Reduction in LVESV by −11.5 ± 10.5% and increased LVEF by 4.8 ± 3.6% (*P* < .001 for both)	Significant improvement in 6MWT and NYHA class noted with CCM
Schau et al,^62^ 2011	54	Median survival 33.1 mo	Retrospective analysis of mortality in patients undergoing CCM	No	—	No difference in survival between CCM patients (median death rate 18.4% per y; 95% CI, 11%-24%) and predicted survival by SHFM (mean 1-y mortality 18.4%; 95% CI, 14.3%-20.1%)	—
Kadish et al,^24^ 2011 (FIX-HF-5)	428	6 mo	CCM plus OMT to CCM alone	Yes	Control	No change in ventilatory anaerobic threshold with CCM (4.5 mL/kg/min; 95% CI, −2.4-11.5; *P* = .314)	Significant improvement in peak VO_2_ and MLWHFQ in CCM over OMT
Kuschyk et al,^43^ 2015	81	34 mo	Single-center analysis of efficacy of CCM	No	Baseline	Lower observed 1-y mortality rate vs predicted rate derived from MAGGIC score (5.2% vs 18.4%; *P* < .001), nonsignificant trend in 3-y mortality rate (29.5% vs 40%)	Significant increase in LVEF, mean NYHA class, and mean peak VO_2_
Kloppe et al,^44^ 2016	68	Mean follow-up of 4.5 y	Survival in CCM patients with NYHA II or II and QRSd ≤130 ms	No	—	Survival at 1, 2, and 5 y was lower with CCM than predicted by SHFM (0% vs 6.1, 3.5% vs 11.8%, and 14.2% vs 27.7%, respectively; *P* = .007)	—
Liu et al,^45^ 2016	82	6 y	Case-control study of survival after CCM vs OMT in HF patients with LVEF ≤40%	No	—	All-cause mortality was lower in CCM group vs control (39% vs 71%; *P* = .001)	—
Kloppe et al,^35^ 2016	19	6 mo	Effect of varying duration of CCM (5 vs 12 h)	Yes	Difference between groups (12h vs 5h)	No significant differences clinically or statistically in MLWHFQ, NYHA, peak VO_2_, 6MWT, or LVEF between groups	—
Röger et al,^46^ 2017	48	6 mo	Comparison of CCM delivery via 2-lead system vs 1-lead system	Yes	Change in each group compared to baseline	Reduction in NYHA class (−0.7±0.5 vs −0.9±0.7; *P* < .05) and MLWHFQ (−14 ± 20 vs −16±22; *P* < .05)	Peak VO_2_ improvement in both groups was not significant
Müller et al,^47^ 2017	143	6, 12, 18, and 24 mo	Prospective registry evaluating the effect of CCM on outcomes by LVEF subgroup	No	Baseline	Improvement in MLWHFQ, NYHA class, and LVEF at 24 mo (*P* < .001 for all)	Overall survival at 24 mo was 86.4% (95% CI, 79.3-91.2)
Abraham et al,^25^ 2018 (FIX-HF-5C)	160	6 mo	Confirm CCM efficacy in patients with LVEF between 25% and 45% in patients with NYHA III-IV	Yes	Control	Increase in peak VO_2_ of 0.84 mL/kg/min (95% Bayesian credible interval 0.123 to 0.1552)	Composite decrease in CV death and HF hospitalizations (10.8% to 2.9%; *P=* .048)
Kuschyk et al,^48^ 2019	17	6 mo	Efficacy of CCM in CRT nonresponders	No	Baseline	Increase in peak VO_2_ (1.1 ± 1.6 mL/kg/min; *P* = .03) and MLWHFQ (−16 ± 6; *P* < .01)	Mean NYHA class and 6MWT also significantly improved
Anker et al,^49^ 2019 (CCM-REG_25-45_)	140	3 y	Prospective registry of CCM in patients with HF and an EF ≤ 45%.	No	Baseline	Improvement in MLWHFQ, NYHA class, and LVEF at 3 y (*P* < .001 for all)	Survival significantly better than predicted by SHFM in patients with LVEF 35-45 (88% vs 74.7%; *P* = .046)
Wiegn et al,^34^ 2020 (FIX-HF-5C2)	60	6 mo	Safety and efficacy of 2-lead CCM system	No	Control group of FIX-HF-5C	Increase in peak VO_2_ of 1.72 mL/kg/min (95% Bayesian credible interval 1.02-2.42)	Decreased CCM-related adverse events with 2-lead system compared with 3-lead system (0% vs 8%; *P* = .03)
Kuschyk et al,^50^ 2021 (CCM-REG)	503	6, 12, 18, and 24 mo	Registry of patients with CCM by LVEF (≤25%, 26%-34%, ≥35%)	No	Baseline (overall cohort)	Improvement in MLWHFQ, NYHA class, and LVEF at 24 mo (*P* < .001 for all)	Estimated survival was significantly better than predicted at 1 and 3 y in the entire cohort. HF hospitalizations significantly decreased in the overall cohort.
Linde et al,^51^ 2022 (CCM-HFpEF)	47	6 mo	Pilot study of CCM in patients with HFpEF (LVEF ≥50%)	No	Baseline	Improvement in KCCQ score (−18 ± 16.6; *P* < .001)	—

6MWT, 6-minute walk time; CCM, cardiac contractility modulation; CRT, cardiac resynchronization therapy; HF, heart failure; HFpEF, heart failure with preserved ejection fraction; KCCQ, Kansas City Cardiomyopathy questionnaire; LVEF, left ventricular ejection fraction; MAGGIC, Meta-Analysis Global Group in Chronic Heart Failure; LVESV, left ventricular end systolic volume; MLWHFQ, Minnesota Living With Heart Failure Questionnaire; NYHA, New York Heart Association; OMT, optimal medical therapy; SHFM, Seattle Heart Failure Model; VO_2_, myocardial oxygen consumption.

The subsequent FIX-HF-5 study was then conducted in a larger cohort of 428 patients with LVEF ≤35%, NYHA class III/IV symptoms despite OMT, ineligibility for CRT, and QRS duration of <130 ms.^24^ Patients were randomized to receive OMT plus CCM (n = 215) vs OMT alone (n = 213). The primary end point of the trial was an improvement in ventilatory anaerobic threshold in the CCM group, which was not met. However, the OMT plus CCM group demonstrated improved quality of life by MLWHFQ, NYHA class, and peak VO_2_ at 6 months, and at 12 months there was comparable all-cause mortality and hospitalizations satisfying a prespecified noninferiority safety end point. A prespecified subgroup analysis revealed that LVEF ≥25% was a predictor of increased efficacy of CCM in the FIX-HF-5 cohort,^53^ prompting the follow-up study FIX-HF-5C. In this randomized trial, 160 symptomatic HF patients with NYHA class III/IV symptoms, LVEF ≥25% and ≤45%, and QRS <130 ms were randomized to OMT plus CCM or continue OMT alone.^25^ At a mean 24 weeks of follow-up, CCM therapy was associated with increased peak VO_2_, and improvement in NYHA functional class, 6-minute walk test, and MLWHFQ score. In addition, a composite end point of cardiovascular death and HF hospitalizations was reduced. Interestingly, in a prespecified subgroup analysis of patients with LVEF ≥35%, a more significant benefit was derived compared to those with LVEF ≥25% but <35%.^25^ The results of FIX-HF-5 and FIX-HF-5C ultimately led to the device gaining FDA breakthrough status in 2019.^54^ The more recent FIX-HF-5C2 study enrolled 60 patients with NYHA III/IV symptoms and LVEF ≥25% and <45% who were not eligible for CRT to test the efficacy and safety of a 2-lead (2 ventricular leads, no atrial lead) Optimizer system.^34^ Results demonstrated comparable device function between the 2-lead and 3-lead system and similar improvement in peak VO_2_ and NYHA class compared to FIX-HF-5C controls. Two important benefits of the 2-lead system were highlighted in this work. First, lead-related adverse events were significantly decreased and second, this system relied solely on sensing from the ventricular leads, allowing CCM therapy to be delivered to patients with AF.

In 2020, Giallauria et al performed a comprehensive individual patient data meta-analysis of all randomized trials, and the nonrandomized FIX-HF-5C2 included 861 patients and pooled analysis showed that CCM significantly improved peak VO_2_, 6-minute walk test distance, and quality of life by MLWHFQ.^55^ Results were similar in a sensitivity analysis excluding the 60 patients from FIX-HF-5C2. The discussion of CCM devices was first included in the European Society of Cardiology HF Guidelines in 2016^56^ and is mentioned in the 2022 ACC/AHA Guidelines for the Management of HF.^5^

In summary, clinical trials both randomized and nonrandomized have demonstrated that CCM therapy in patients with HFrEF and HFmrEF in addition to OMT improves peak VO_2_, 6-minute walk test, NYHA functional class, and quality of life. The patients who seem to benefit most are those with LVEF between 25% and 45% and NYHA class II-III.^25^^,^^29^ A positive effect of CCM on cardiovascular outcomes including mortality and hospitalizations is suggested by some randomized trials and retrospective studies,^25^^,^^44^, ^45^, ^46^^,^^49^ but requires verification and further study in prospective, randomized controlled trials.

## Future directions

Accruing evidence suggests that CCM may fill the gap in device-based HF therapy for patients with HFrEF and HFmrEF who are not candidates for CRT or are CRT nonresponders. The available evidence comes from modestly sized trials with relatively short duration of follow-up, and it remains unknown whether there is an improved benefit over longer time periods and whether CCM increases LVEF, exerts effects on reverse remodeling, and improves cardiovascular outcomes. Importantly, increases in peak VO_2_ have been shown to improve clinical outcomes in HF patients,^57^ and it is possible that the improvement in peak VO_2_ with CCM may signify that better cardiovascular outcomes will be observed in larger prospective studies.

Technological advances are likely to increase the feasibility, compatibility, and safety profile of CCM in HF patients, especially those who require additional indwelling cardiac devices (eg, PPM or ICD). One randomized study of 48 patients assessed the safety and efficacy of CCM therapy delivered through 1 vs 2 leads and showed similar improvement in NYHA class, MLWHFQ score, and Peak VO_2_.^46^ Decreasing the number of leads required may reduce the risk of infection and tricuspid regurgitation as well as venous occlusion. Furthermore, combination devices may further reduce the need for multiple leads, indwelling generators, and procedures. Recently, the first combined CCM-ICD device (Optimizer Integra CCM-D system) was implanted,^58^ and a multicenter trial (INTEGRA-D) investigating this therapy is enrolling.

Recently, a pilot study (CCM-HFpEF) was conducted in 47 patients with HFpEF and NYHA class II or III symptoms demonstrated an improvement in Kansas City Cardiomyopathy Questionnaire (KCCQ) summary score,^51^^,^^59^ suggesting that the benefits of CCM may extend to the growing population of patients with HFpEF. The ongoing AIM HIGHer Clinical Trial (NCT 05064709) is a prospective, multicenter randomized controlled trial in patients with LVEF ≥40% and ≤60% and is expected to enroll 1500 participants with an estimated completion date in 2026, and will lend valuable insight into the role of CCM in this population.^60^ In addition, a recent review by Riccardi et al expands upon the rationale and role of CCM in patients with HFpEF, highlighting the potential impact of CCM therapy on calcium homeostasis, reverse remodeling, and favorable clinical outcomes after CCM therapy in patients with HFpEF.^61^

Accruing evidence suggests that CCM may be beneficial among patients with HF across a broad range of LVEF. However, the mechanism through which CCM exerts its beneficial effects remains incompletely understood and is an area for future study. In addition, despite evidence that CCM improves outcomes such as quality of life, NYHA class, and peak VO_2_, much of the data comes from meta-analyses and secondary end points of randomized controlled trials. More studies are needed to determine whether CCM therapy improves long-term outcomes including mortality, cardiovascular death, and HF hospitalizations.

## Conclusion

CCM represents a unique device-based therapy for patients with HF. There is robust evidence to suggest improved quality of life and functional status with CCM, and future trials are needed to confirm benefits on mortality, hospitalization, and the use of CCM in patients with HFpEF. CCM should be considered in patients with HFrEF and HFmrEF who have a narrow QRS complex and are symptomatic despite OMT.
